# Resilient Control Under Side-Channel Compromise for IoMT Devices

**DOI:** 10.3390/s26195987

**Published:** 2026-09-22

**Authors:** Mordecai Opoku Ohemeng, Frederick T. Sheldon

**Affiliations:** Department of Computer Science, University of Idaho, Moscow, ID 83844, USA; sheldon@uidaho.edu

**Keywords:** microarchitectural leakage, Control Barrier Function, Quadratic Programming, Internet of Medical Things

## Abstract

As Internet of Medical Things (IoMT) ecosystems expand, life-critical devices become increasingly vulnerable to microarchitectural leakage attacks that enable adversaries to evade conventional digital authentication mechanisms. Exploiting timing and cache leakage allows adversaries to extract credentials and issue cryptographically valid commands with physiologically unsafe parameters, a systemic weakness we define as the verified attacker problem. This work introduces a dual-layer physiological and side-channel defense architecture that maintains patient safety even under credential compromise. At the perception layer, single-lead 1D electrocardiogram (ECG) telemetry is restored using a 1D Denoising Autoencoder (1D-DAE) to counteract hardware perturbation proxies: actuation jitter, cache eviction block erasures, and password verification timing interference. The 1D-DAE maintains high signal reconstruction fidelity (PSNR≈29.1–36.7dB, SSIM≈0.81–0.89), stabilizing diagnostic classification accuracy above 90% across all perturbation conditions. At the actuation layer, a Control Barrier Function (CBF) filter enforces physical safe-set invariance, instantly projecting a malicious pacing command injection ($u_{\mathrm{req}}=300\;\mathrm{BPM}$) down to a safe 140BPM bound using real-time Quadratic Programming (QP). Hardware profiling yields an end-to-end execution latency of 2.09ms and a low 7.2mJ energy footprint per cycle, confirming real-time feasibility for continuous 360Hz telemetry monitoring and safe closed-loop pacing.

## 1. Introduction

### 1.1. Background

Combining Artificial Intelligence (AI) with Internet of Medical Things (IoMT) technology allows life-critical devices, such as cardiac pacemakers, automated insulin pumps, and neurostimulators, to continuously track patient health and automatically administer care in real time [1]. However, connecting these devices to digital networks introduces severe safety risks [2]. Healthcare cyberattacks have increased by 55% in recent years [3], with emerging trends showing that threats are increasingly aimed at manipulating physical medical device control loops rather than merely breaching patient privacy [4]. Standard cybersecurity focuses on the CIA triad (Confidentiality, Integrity, and Availability). For life-critical devices, safety requires physiological stability [5]. Modern networked feedback systems face complex propagation dynamics; for instance, resilient estimation and adaptive control schemes must explicitly balance safety guarantees against usability constraints when managing cascading disruptions over networked infrastructures [6]. Current regulations often fail to safeguard AI-enabled medical devices, where hardware leaks, signal losses, or malicious inputs can cause dangerous treatment errors that bypass traditional network defenses. To improve safety, we introduce a dual-layer protection system that pairs 1D Convolutional Denoising Autoencoder (DAE) signal restoration with Control Barrier Function (CBF) optimization [7]. This framework keeps device behavior within safe patient limits, even when a cyberattack is under way.

### 1.2. Characterization of IoMT Vulnerabilities

We have grouped security risks in connected IoMT systems into three: hardware and microarchitectural vulnerabilities, software and network protocol weaknesses, and control-theoretic threats to physiological safety [8,9].

#### 1.2.1. Hardware-Layer and Microarchitectural Vulnerabilities

At the hardware layer, vulnerabilities arise from how processors execute instructions. Attackers can exploit non-constant-time operations [10], shared cache behavior [11,12], or power fluctuations on system buses to infer secret keys or recover timing information [13]. Microarchitectural events such as CPU stalls during password verification or cache contention during cryptographic operations introduce physical distortions into streaming telemetry. The work in [14] showed that hardware-level feature perturbations cause diagnostic accuracy to drop by over 18% in embedded signal processing pipelines. While offline techniques such as Sharpness-Aware Minimization reduce noise sensitivity during training, they cannot monitor devices in real time or block side-channel attacks during active operation.

#### 1.2.2. Software and Network Protocol Weaknesses

The software protocol includes implementation bugs [15], weak authentication [16], and slow update cycles [17]. Audits show that over 50% of active medical hardware contains known software vulnerabilities, remaining unpatched for an average of 3.2 years [9]. Software diagnostics also suffer performance drops over time due to natural physiological baseline shifts [18]. While traditional approaches often rely on architectures like Multi-Head Self-Attention LSTMs, contemporary paradigms have shifted toward neural network-based estimation and control of cyber-physical systems using artificial intelligence [19] to bolster overall framework security. Stochastic-Aware Blockchain Controllers [20] and physics-constrained optimization frameworks for detecting stealthy drift perturbations [21] provide robust detection of network anomalies and physical data injection attempts.

However, these software-centric defenses become ineffective when an adversary acquires legitimate credentials through hardware leakage [22,23], allowing malicious commands to be issued over fully authorized communication paths without triggering anomaly-based detection [24,25,26].

#### 1.2.3. Control-Theoretic Threats to Physiological Safety

The final layer is the patient’s physiological system itself, yet most IoMT controllers provide no formal safety verification, exposing critical functions to uncontrolled or unsafe actuation. High-accuracy deep learning models perform well under clean conditions [27], but they do not account for physical biological limits. When embedded classifiers face telemetry distortions, such as actuation jitter, cache eviction signal drops, or timing interference, reconstruction models optimizing standard Mean Squared Error (MSE) can generate non-physiological cardiac waveforms. Furthermore, computational resource and execution delays can degrade closed-loop timing boundaries unless governed by control-theoretic guarantees [28,29,30]. Without such control-theoretic guarantees, degraded diagnostic accuracy allows malicious pacing commands to reach the patient without constraint.

### 1.3. Key Safety Gaps

Current IoMT security architectures suffer from three critical gaps:

1. Stolen credentials allow attackers to bypass software authentication. Existing diagnostic models adjust decision boundaries statistically [31] but lack hard, deterministic safety overrides to prevent physiologically unsafe actuation.
2. Network intrusion detection systems and adaptive networked controllers [6] inspect packet patterns or estimation dynamics rather than hard physiological patient bounds. They cannot stop cryptographically valid commands that deliver unsafe therapy.
3. Standard Denoising Autoencoders optimize mathematical reconstruction loss (MSE) rather than control-invariant safety boundaries, offering no guarantees that restored telemetry will remain within acceptable clinical thresholds.

### 1.4. Key Contributions

This paper makes the following key contributions:

1. It presents an integrated framework pairing 1D Convolutional Denoising Autoencoder (DAE) signal restoration with real-time Control Barrier Function (CBF) Convex Quadratic Program (QP) filtering to maintain physiological safety under credential misuse and telemetry distortion.
2. It models three realistic microarchitectural hardware perturbations on real PhysioNet MIT-BIH Lead MLII telemetry (Subject 100): Actuation Jitter ($V_{\mathrm{time}}$), Cache Eviction Telemetry Loss ($V_{\mathrm{cache}}$), and Password Timing Interference ($V_{\mathrm{auth}}$), demonstrating recovery of diagnostic accuracy above a 90% clinical threshold along with elevated PSNR and SSIM quality metrics.
3. It formalizes a parameter-driven CBF-QP filter enforcing strict physiological state invariance ($u_{\max}=140\;\mathrm{BPM}$) under active command injection attacks.
4. It profiles the five-stage execution latency (ms) and energy footprint across preprocessing, DAE restoration, CNN classification, CVXPY QP filtering, and I/O output under a 3.5W power envelope, confirming real-time deployment feasibility for 360Hz IoMT telemetry processing.

## 2. Model

The interaction between the medical device and the patient is modeled as a continuous-time control-affine dynamical system. Let x(t)∈R denote the physiological state variable (heart rate in BPM), and let u(t)∈R denote the therapeutic pacing command. The plant dynamics evolve according to

(1) $$\dot{x}\left(t\right)=f\left(x\left(t\right)\right)+g\left(x\left(t\right)\right)\left(u\left(t\right)-x_{\mathrm{base}}\right)+\omega \left(t\right),$$

where $\omega \left(t\right)∼N\left(0,\sigma^{2}\right)$ represents stochastic biological variability. The natural metabolic drift field f(x(t)) is modeled as a first-order relaxation process:

(2) $$f\left(x\left(t\right)\right)=-\alpha \left(x\left(t\right)-x_{\mathrm{base}}\right),$$

with nominal resting cardiac baseline $x_{\mathrm{base}}=75.0\;\mathrm{BPM}$, tissue recovery decay rate $\alpha =0.8\;s^{-1}$, and constant actuation coupling gain g(x(t))=β=0.8.

**Remark** **1.** *We maintain a strict distinction between the requested control command u(t) issued by the device firmware and the continuous physical state response x(t) of the patient tissue. Pacing commands dictate target electrical pulse rates, whereas the physical state variable x(t) evolves continuously according to* (1) *and* (2).

### 2.1. Stage 1

Following established paradigms in simulation-based fault injection and cyber-physical robustness evaluation [32,33], we model microarchitectural execution contention through continuous perturbation proxies ($V_{\mathrm{time}},V_{\mathrm{cache}},V_{\mathrm{auth}}$). Grounding these proxies in analytical models of hardware memory bus delays and execution jitter [34,35] enables systematic benchmarking of the perception restoration pipeline under repeatable, worst-case degradation bounds before CBF safety enforcement.

We model hardware vulnerabilities arising within the microprocessor execution boundary. Execution contention on resource-constrained microcontrollers directly impacts the Analog-to-Digital Converter (ADC) interface and Direct Memory Access (DMA) telemetry buffers. We model these impacts as three discrete temporal perturbation operators applied to normalized 1D ECG telemetry signals $x\in R^{1\times N}$ (N=720 samples at $f_{s}=360\;\mathrm{Hz}$):

- Actuation Jitter Perturbation ($V_{\mathrm{time}}$): Execution-time variance disrupts ADC sampling regularity. At $f_{s}=360\;\mathrm{Hz}$ (Δt≈2.78ms), variable interrupt latency and task scheduling jitter (typically 100–400μs on embedded microcontrollers) are manifested as sampling phase noise and voltage translation errors. In normalized telemetry, this is modeled as additive zero-mean Gaussian noise scaled by $\sigma_{\mathrm{jitter}}=0.15$, establishing a worst-case noise floor (≈15% SNR degradation) to bound timing uncertainty:
  (3) $$x_{\mathrm{jitter}}\left(t\right)=x\left(t\right)+\eta \left(t\right),\;\eta \left(t\right)∼N\left(0,0.15^{2}\right).$$
- Cache-Contention Telemetry Erasure ($V_{\mathrm{cache}}$): Heavy memory bus locking and cache eviction loops during administrative execution stall DMA buffer transfers on low-power architectures (ARM Cortex-M running at 16–48MHz). This causes a burst loss of telemetry data, modeled as a zero mask across $k_{1}=220$ to $k_{2}=340$ (120 consecutive samples at 360Hz, corresponding to a Δt≈0.33s DMA stall spanning a full cardiac depolarization event):
  (4) $$x_{\mathrm{cache}}\left[k\right]=\left\{\begin{array}{cc} 0.0 & \mathrm{for}\;220\leq k\leq 340,\\ x[k] & \mathrm{otherwise}. \end{array}\right.$$
- Password Verification Timing Interference ($V_{\mathrm{auth}}$): Non-constant-time cryptographic loops during password verification draw periodic supply-current spikes, inducing voltage ripple on internal lines. We set the interference frequency to $f_{\mathrm{auth}}=35.0\;\mathrm{Hz}$ and amplitude to $A_{\mathrm{auth}}=0.35$ because 35.0Hz falls directly within the primary diagnostic frequency band of ECG signals (0.5–40Hz). This tests whether the restoration model can decouple physical supply-line side-channel interference from legitimate physiological QRS/T-wave morphology:
  (5) $$x_{\mathrm{auth}}\left(t\right)=x\left(t\right)+A_{\mathrm{auth}}\sin\left(2\pi f_{\mathrm{auth}}t\right).$$

Raw time-series windows are zero-mean unit-variance normalized ($\tilde{x}\left[k\right]=\frac{x\left[k\right]-\mu_{x}}{\sigma_{x}+\epsilon }$) and passed through the 1D Denoising Autoencoder (1D-DAE) to map distorted telemetry back onto the ground-truth physiological manifold before downstream classification.

### 2.2. Stage 2

Once an adversary exfiltrates administrative credentials via Stage 1 vulnerabilities, that party can issue authenticated, malicious pacing commands $u_{\mathrm{req}}\left(t\right)$ that bypass network signature validation. To evaluate the active CBF supervisor under worst-case operational failure, we model the requested command as an unconstrained, synthetic actuator saturation step injection $u_{\mathrm{req}}\left(t\right)=300.0\;\mathrm{BPM}$. Here, 300.0BPM serves as an extreme hardware benchmark bound, representing a worst-case malicious software payload or firmware override at the digital interface, rather than a plausible biological state.

To guarantee physical safety despite complete credential compromise, we define the physiologically safe set

(6) C={x∈R∣h(x)≥0},

using the candidate Control Barrier Function (CBF):

(7) $$h\left(x\right)=x_{\max}-x\left(t\right),$$

where $x_{\max}=140.0\;\mathrm{BPM}$ represents the maximum allowable physiological heart rate defined by clinical guidelines [36] to prevent acute tachyarrhythmia, hemodynamic instability, and myocardial strain in adult cohorts.

Enforcing forward invariance of C, the safety filter enforces the dynamic rendering condition $\dot{h}\left(x,u\right)\geq -\gamma h\left(x\right)$, where $\gamma =0.8\;s^{-1}$ dictates the linear class-K convergence rate. Expanding the time derivative along system dynamics yields

(8) $$-\left(-\alpha \left(x-x_{\mathrm{base}}\right)+\beta \left(u-x_{\mathrm{base}}\right)\right)\geq -\gamma \left(x_{\max}-x\right),$$

which simplifies to the following linear inequality constraint on the control input u(t):

(9) $$\beta \left(u-x_{\mathrm{base}}\right)\leq \alpha \left(x-x_{\mathrm{base}}\right)+\gamma \left(x_{\max}-x\right).$$

When an adversarial command $u_{\mathrm{req}}\left(t\right)=300.0\;\mathrm{BPM}$ is received, the active Control Barrier Function-Quadratic Program (CBF-QP) supervisor solves

(10) $$\begin{array}{cc} u_{\mathrm{final}}\left(t\right)=\arg\min_{u\in R}\; & \frac{1}{2}{\left(u-u_{\mathrm{req}}\left(t\right)\right)}^{2}\\ s.t.\; & 0.8(u-75.0)\leq 0.8(x-75.0)+0.8(140.0-x). \end{array}$$

When $u_{\mathrm{req}}\left(t\right)$ violates inequality (9), the QP filter projects the command down to $u_{\mathrm{final}}\left(t\right)=140.0\;\mathrm{BPM}$, preventing supraventricular over-pacing and guaranteeing forward invariance of C.

### 2.3. The Proposed Framework

To neutralize the cross-layer threat introduced by credential compromise and hardware interference, the framework implements a sequential two-layer defense architecture. Layer 1 mitigates raw telemetry corruption arising from hardware-induced perturbations ($V_{\mathrm{time}},V_{\mathrm{cache}},V_{\mathrm{auth}}$), while Layer 2 enforces formal control-theoretic safety constraints directly on the physiological actuation loop.

#### 2.3.1. Layer 1

Layer 1 restores corrupted 1D temporal telemetry streams and identifies anomalous physiological patterns. A 1D Denoising Autoencoder (1D-DAE), denoted by R, acts as a non-linear manifold projection operator mapping the corrupted time-series window $x_{\mathrm{sig}}\in R^{1\times 720}$ onto the nominal physiological manifold:

(11) $$\hat{x}=R\left(x_{\mathrm{sig}}\right)=f_{\theta }\left(g_{\phi }\left(x_{\mathrm{sig}}\right)\right),$$

where $g_{\phi }$ (two convolutional layers with batch normalization and ReLU activations) and $f_{\theta }$ (two transposed convolutional layers) represent the encoder and decoder networks.

Reconstruction performance on 1D temporal signals is evaluated using Peak Signal-to-Noise Ratio (PSNR) and Structural Similarity Index Measure (SSIM with window size w=7):

(12) $$\mathrm{PSNR}=20\log_{10}\left(\frac{\max\left(x_{\mathrm{clean}}\right)-\min\left(x_{\mathrm{clean}}\right)}{\sqrt{\frac{1}{L}\sum_{k=1}^{L}{\left(x_{\mathrm{clean}}\left[k\right]-\hat{x}\left[k\right]\right)}^{2}}}\right),$$

(13) $$\mathrm{SSIM}\left(x,\hat{x}\right)=\frac{\left(2\mu_{x}\mu_{\hat{x}}+c_{1}\right)\left(2\sigma_{x\hat{x}}+c_{2}\right)}{\left(\mu_{x}^{2}+\mu_{\hat{x}}^{2}+c_{1}\right)\left(\sigma_{x}^{2}+\sigma_{\hat{x}}^{2}+c_{2}\right)}.$$

Empirical evaluation across 100 segmented windows extracted from records 100, 101, 102, and 103 of the PhysioNet MIT-BIH Arrhythmia Database confirms high-fidelity waveform restoration. The reconstructed signal window $\hat{x}$ is subsequently processed by a 1D Convolutional Neural Network (1D-CNN) classifier consisting of 1D convolutions, batch normalization, ReLU, and adaptive average pooling to evaluate diagnostic classification in clean and corrupted states.

#### 2.3.2. Layer 2

Layer 2 provides continuous physical safety guarantees by enforcing hard constraints on the physiological control loop. We maintain forward invariance of the closed safe operating set C by solving the minimal-norm convex QP optimization in (10) at each control step. When an adversary uses stolen credentials to issue an unconstrained step command $u_{\mathrm{req}}\left(t\right)=300.0\;\mathrm{BPM}$, the CBF-QP filter projects the command to a boundary-clamped input $u_{\mathrm{final}}\left(t\right)=140.0\;\mathrm{BPM}$, ensuring that the physiological heart-rate trajectory x(t) smoothly approaches 140.0BPM without violating the safety boundary (h(x)≥0).

### 2.4. Attack Model

We model the microarchitectural leakage exploited in Stage 1 as a strictly passive observation channel that reveals information about secret-dependent execution behavior without directly altering continuous cardiac tissue dynamics. Let the device’s execution time *T* and instantaneous power consumption P(t) be functions of the secret key *k* and input data *x*. We focus on evaluating framework resilience against downstream physical telemetry perturbations induced by side-channel execution contention, rather than on-chip key extraction mechanics. To achieve controlled, reproducible benchmarking across extreme operating regimes, microarchitectural contention effects are captured via analytical perturbation models ($V_{\mathrm{time}},V_{\mathrm{cache}},V_{\mathrm{auth}}$). These models serve as empirically grounded proxies representing the physical signal manifestations of hardware contention on ADC sampling and DMA transfers.

#### 2.4.1. Timing Leakage and Actuation Jitter

Following Kocher’s timing attack model [37], the total execution time of a non-constant-time cryptographic routine is expressed as

(14) $$T=\sum_{i=0}^{N}t_{i}\left(k_{i},x_{i}\right),$$

where $t_{i}$ denotes the key- and data-dependent latency of the *i*th operation. An adversary measuring *T* over repeated queries can statistically infer private credentials; while trace analysis enables key reconstruction, the accompanying processor execution contention disrupts Analog-to-Digital Converter (ADC) sampling regularity. On 1D Lead MLII ECG telemetry streams sampled at $f_{s}=360\;\mathrm{Hz}$, this is modeled as the Actuation Jitter perturbation ($V_{\mathrm{time}}$):

(15) $$x_{\mathrm{jitter}}\left[k\right]=x\left[k\right]+\eta \left[k\right],\;\eta \left[k\right]∼N\left(0,0.15^{2}\right).$$

#### 2.4.2. Power and Cache-Contention Leakage

Power consumption is modeled using the Hamming-weight leakage formulation from differential power analysis (DPA) [38]. Let v(t) denote an intermediate register value during computation:

(16) P(t)=α·HW(v(t))+ξ(t),

where HW(·) is the Hamming weight, α is a scaling constant, and ξ(t) represents electronic noise. Similarly, Flush+Reload L3 cache-contention attacks [39] monitor cache line evictions.

Memory bus contention during trace collection stalls Direct Memory Access (DMA) transfer threads, yielding the cache eviction telemetry loss perturbation ($V_{\mathrm{cache}}$) where a zero mask is applied across samples 220≤k≤340 (120 consecutive samples, Δt≈0.33s):

(17) $$x_{\mathrm{cache}}\left[k\right]=\left\{\begin{array}{cc} 0.0 & \mathrm{for}\;220\leq k\leq 340,\\ x[k] & \mathrm{otherwise}. \end{array}\right.$$

Additionally, non-constant-time string comparison loops during administrative authentication induce high-frequency supply-line ripple. This is modeled as the password verification timing interference perturbation ($V_{\mathrm{auth}}$) via 35Hz sinusoidal superposition:

(18) $$x_{\mathrm{auth}}\left[k\right]=x\left[k\right]+0.35\sin\left(2\pi \cdot 35\cdot t\left[k\right]\right).$$

These microarchitectural leakage channels induce discrete hardware perturbations ($V_{\mathrm{time}},V_{\mathrm{cache}},V_{\mathrm{auth}}$) across the normalized 1D telemetry stream (N=720). Once administrative credentials are successfully exfiltrated via side-channel analysis, the adversary acquires authorized access to inject malicious step-command pacing requests ($u_{\mathrm{req}}\left(t\right)=300.0\;\mathrm{BPM}$) in Stage 2.

### 2.5. The Verified Attacker Problem

When passive microarchitectural monitoring gives way to active interference within the control loop, a systemic cyber-physical vulnerability emerges, which we designate as the verified attacker problem.

The microarchitectural timing and cache contention artifacts modeled in Stage 1 are passive execution-level behaviors (execution-time variance, cache evictions, supply-line ripple) that leak information during cryptographic routines. Once administrative credentials are exfiltrated via side-channel analysis, the adversary transitions from passive observation to active manipulation.

Equipped with valid administrative credentials, the attacker signs a malicious pacing command $u_{\mathrm{req}}\left(t\right)$ that is cryptographically indistinguishable from legitimate clinical instructions. Because conventional IoMT defenses rely primarily on digital signatures and packet-integrity validation [8], these forged commands bypass network-layer security perimeters and pass directly to the device actuation loop. By issuing an unconstrained step command $u_{\mathrm{req}}\left(t\right)=300.0\;\mathrm{BPM}$, the adversary attempts to force the continuous physiological state x(t) into the unsafe physiological set

(19) U={x∈R∣h(x)<0},

inducing acute hyperdynamic supraventricular tachycardia.

To resolve the verified attacker problem, the proposed framework shifts verification from digital trust (signatures and packet headers) to physical-layer telemetry restoration and real-time control safety projection. Rather than rely on cryptographic metadata alone, the architecture evaluates whether the restored telemetry signal $\hat{x}$ and requested control input $u_{\mathrm{req}}\left(t\right)$ satisfy clinical safety bounds.

#### Telemetry Restoration and Anomaly Detection

Prior to classification, the 1D-DAE filters out hardware-induced perturbations ($V_{\mathrm{time}},V_{\mathrm{cache}},V_{\mathrm{auth}}$), restoring normalized 1D ECG telemetry signals ($x_{\mathrm{sig}}\in R^{1\times 720}$). Benchmarking across 100 segmented evaluation windows (N=720 at $f_{s}=360\;\mathrm{Hz}$) from PhysioNet MIT-BIH Lead MLII records confirms consistent morphological PQRST restoration.

This high-fidelity reconstruction allows the downstream 1D-CNN classifier to evaluate diagnostic classification and anomaly posterior probabilities $P(A|\hat{x})$ without interference from 120-sample zeroed cache dropouts or high-frequency side-channel noise. Once an anomaly is detected ($D(x_{\mathrm{sig}})=1$), the framework engages the active CBF filter to guarantee that the physiological state remains within the safe set C (6).

When an authenticated adversary requests an unconstrained step command $u_{\mathrm{req}}\left(t\right)=300.0\;\mathrm{BPM}$, the plant dynamics evolve according to (1) and (2). To prevent this command from driving x(t) into U, the CBF-QP supervisor solves the minimal-norm convex optimization program in (10).

When $u_{\mathrm{req}}\left(t\right)=300.0\;\mathrm{BPM}$ violates condition (9), the QP filter projects the command to the safe, boundary-clamped input $u_{\mathrm{final}}\left(t\right)=140.0\;\mathrm{BPM}$. Enforcing this constraint guarantees that the safe boundary ∂C={x∈R∣h(x)=0} is rendered forward invariant. Consequently, for any initial state x(0)∈C, the physiological state trajectory x(t) smoothly converges toward 140.0BPM without violating h(x)≥0, nullifying the threat of authenticated adversarial actuation.

## 3. Experiment

### 3.1. Experimental Setup

Evaluating closed-loop IoMT security systems in real-world clinical environments is challenging due to the restricted availability of live patient telemetry and regulatory mandates imposed by HIPAA. To ensure strict empirical reproducibility without violating patient privacy laws, we utilize publicly available clinical ECG telemetry from the PhysioNet MIT-BIH Arrhythmia Database (Lead MLII telemetry, Records 100, 101, 102, and 103) [40]. Data processing scripts and complete pipeline implementation code are publicly available at: https://github.com/opoku-ohemeng/IoMT-compromise (accessed on 16 September 2026).

Raw 360Hz single-lead ECG signals are partitioned into non-overlapping 2 s windows (L=720 continuous voltage samples per window, with 25 segments per record yielding N=100 total evaluation windows). Our framework processes 1D temporal vectors directly to maintain ultra-low latency and computational efficiency on embedded microcontrollers.

To stress-test the robustness of the detection and safety pipeline against cross-layer hardware-induced telemetry degradation, we introduce three controlled perturbation models that approximate physical hardware artifacts resulting from execution contention and side-channel leakage:

1. Actuation Jitter ($V_{\mathrm{time}}$): Models additive zero-mean Gaussian sampling jitter induced by CPU execution stalls during non-constant-time cryptographic processing:
  (20) $$x_{\mathrm{corrupted}}\left[k\right]=x\left[k\right]+\eta \left[k\right],\;\eta \left[k\right]∼N\left(0,0.15^{2}\right).$$
2. Cache-Eviction Telemetry Loss ($V_{\mathrm{cache}}$): Introduces a zero-mask across samples 220≤k≤340 (120 consecutive samples, Δt≈0.33s) to emulate DMA buffer drops caused by Flush+Reload cache eviction miss penalties:
  (21) $$x_{\mathrm{corrupted}}\left[k\right]=\left\{\begin{array}{cc} 0.0 & \mathrm{for}\;220\leq k\leq 340,\\ x[k] & \mathrm{otherwise}. \end{array}\right.$$
3. Password Verification Timing Interference ($V_{\mathrm{auth}}$): Applies high-frequency periodic harmonic sinusoidal waves to model supply-line voltage ripple arising during non-constant-time string comparisons:
  (22) $$x_{\mathrm{corrupted}}\left[k\right]=x\left[k\right]+0.35\sin\left(2\pi \cdot 35\cdot t\left[k\right]\right).$$

These perturbations model real physical cross-layer signal degradation occurring at the microcontroller ADC and DMA buffer boundary.

**Remark** **2.** *Following standard validation practices for cross-layer cyber-physical resilience, these perturbation models serve as analytical proxies isolating the physical manifestation of microarchitectural interference on analog telemetry, rather than executing live cryptographic extraction primitives on physical microcontrollers. This controlled formulation parameterizes the perturbations (σ=0.15, 0.33s DMA stall, 35Hz diagnostic-band ripple) to reflect empirical worst-case operational bounds, enabling reproducible benchmarking of perception restoration accuracy under severe signal degradation independent of specific silicon microarchitectures or physical bus layouts.*

A 1D-DAE acts as the primary restoration engine, passing cleansed 1D time-series streams to a lightweight 1D-CNN classifier and downstream convex CBF-QP safety filter.

### 3.2. Implementation Parameters and Execution Profiling Setup

The complete pipeline, comprising the 1D-DAE signal restoration model, 1D-CNN classifier, and OSQP-backed CBF-QP safety filter, was implemented in PyTorch v. 2.13.0 and executed within a Python v. 3.10 runtime environment in Google Colab. Table 1 summarizes the core hyperparameter configurations, network architectures, and dataset splits.

#### Computational Latency Profiling Methodology

To evaluate the real-time processing overhead of the closed-loop defense pipeline, stage-by-stage execution latency was profiled directly within the execution environment across 10,000 continuous 360Hz ECG window execution cycles (L=720 samples). Micro-benchmarking was conducted using high-precision hardware timers (time.perf_counter() for CPU execution and CUDA event synchronization primitives torch.cuda.Event() for neural network inferences) to eliminate asynchronous queuing overheads and capture pure per-stage execution times.

### 3.3. Evaluation of Telemetry Restoration and CBF Safety Enforcement

This experiment evaluates the proposed framework across the three cross-layer perturbation models ($V_{\mathrm{time}}$, $V_{\mathrm{cache}}$, and $V_{\mathrm{auth}}$), measuring signal restoration quality, diagnostic classification performance, and real-time CBF safety enforcement.

#### 3.3.1. Signal Restoration and Diagnostic Classification

Layer 1 processes 1D ECG telemetry streams through the 1D-DAE. The 1D-DAE maintains high waveform reconstruction quality across all evaluated hardware perturbation vectors:

- ($V_{\mathrm{time}}$): PSNR≈36.7dB,SSIM≈0.89.
- ($V_{\mathrm{cache}}$): PSNR≈29.1dB,SSIM≈0.81.
- ($V_{\mathrm{auth}}$): PSNR≈34.7dB,SSIM≈0.83.

As shown in Figure 1, raw corrupted signals severely degrade 1D-CNN diagnostic classification accuracy. The 1D-DAE restoration recovers classification performance back above the 90.0% clinical acceptability threshold across all perturbation scenarios, ensuring reliable anomaly detection under hardware-level side-channel noise.

#### 3.3.2. CBF Safety Layer Validation

Evaluating the CBF safety supervisor under severe adversarial failure modes, we simulate a worst-case unconstrained step-command injection ($u_{\mathrm{req}}\left(t\right)=300.0\;\mathrm{BPM}$) at the digital actuator interface. Here, 300.0BPM serves as a synthetic benchmark parameter representing maximum hardware pulse frequency/command saturation to stress-test instantaneous constraint filtering, rather than a plausible biological state. The safe physiological boundary is defined at $x_{\max}=140.0\;\mathrm{BPM}$, reflecting standard clinical upper limits for cardiac pacing in adult populations to prevent myocardial ischemia and hemodynamic distress during resting/moderate exertion. When the attack payload is injected, the active CBF filter solves the convex QP in real time using (9) and (10).

As shown in Figure 2, the active supervisor clamps the requested control input to $u_{\mathrm{final}}\left(t\right)=140.0\;\mathrm{BPM}$, enforcing forward invariance of the safe operating set C={x∈R∣h(x)≥0} with upper bound $x_{\max}=140.0\;\mathrm{BPM}$ ($\gamma =0.8\;s^{-1}$). This prevents acute hyperdynamic supraventricular tachycardia despite complete credential compromise.

#### 3.3.3. Edge Hardware Execution Profiling

To verify deployment feasibility under a 3.5W nominal power footprint, all five execution stages were micro-benchmarked, as illustrated in Figure 3:

- Stage 1: Preprocess Embedding (0.04ms,0.0001J). Normalizes raw 1D time-series windows (L=720) for pipeline ingestion.
- Stage 2: 1D-DAE Restoration (0.42ms,0.0015J). Removes hardware-induced timing, cache, and authentication perturbations.
- Stage 3: Classifier Inference (0.30ms,0.0010J). Performs diagnostic anomaly inference.
- Stage 4: CBF-QP Filter (1.33ms,0.00460J). Solves the OSQP convex optimization to enforce physiological forward invariance.
- Stage 5. SPI Hardware I/O (0.00ms,0.0000J): Formats and transmits verified pacing commands.

The cumulative end-to-end processing latency is 2.09ms with a total energy consumption of 7.20mJ (0.00720J) per execution cycle. This comfortably satisfies the 2.78ms real-time closed-loop processing deadline for 360Hz ECG telemetry monitoring.

### 3.4. Inter-Patient Generalization and Telemetry Restoration Analysis

We conducted a Leave-Record-Out (LRO) inter-patient cross-validation experiment. As illustrated in Figure 4a, the pipeline was evaluated using a strict 4-fold cross-validation protocol across MIT-BIH records 100, 101, 102, and 103. In each iteration, three continuous patient records served as the reconstruction training suite, while the remaining unobserved patient record was reserved exclusively for evaluation under concurrent cross-layer perturbations ($V_{\mathrm{time}},V_{\mathrm{cache}},V_{\mathrm{auth}}$).

Across all unobserved test records, the 1D-DAE achieved an average reconstruction fidelity of PSNR=26.79±1.81dB (MSE=0.2245). Individual record performance remained consistently robust, yielding 28.71dB on Record 100, 27.88dB on Record 101, and 26.64dB on Record 103. A minor performance dip was observed on Record 102 (PSNR=23.93dB), which is attributed to the presence of paced cardiac rhythms featuring high-frequency artificial pacing spikes and inverted QRS morphologies unobserved in the normal sinus rhythm training sets. Despite these morphological anomalies, the pipeline maintained stable signal restoration without catastrophic divergence, confirming strong cross-subject generalizability.

Figure 4b highlights a representative 2 s telemetry window from unobserved Record 100 under simultaneous attack conditions. The corrupted telemetry stream exhibits severe distortion: continuous actuation jitter (σ=0.15), a persistent 35Hz supply-rail harmonic ripple, and a complete 120-sample zeroing window (t∈[0.61s,0.94s]) induced by DMA execution stalls during cache eviction loops. As demonstrated by the restored output curve, the 1D-DAE effectively decouples high-frequency power-line harmonics from genuine QRS complex depolarizations while generating a smooth, clinically meaningful neural interpolation through the missing DMA buffer interval. This restored signal manifold serves as a high-fidelity input to downstream safety filters, ensuring accurate evaluation under adversarial operation.

## 4. Discussion

The empirical results of this study demonstrate the core of the verified attacker problem in IoMT security: digitally authenticated commands do not guarantee physiologically safe outcomes. By combining microarchitectural leakage analysis with control-theoretic safety enforcement, the proposed framework maintains patient safety even when cryptographic trust is compromised.

### 4.1. Cross-Layer Telemetry Distortion Analysis

As demonstrated across the online clinical telemetry evaluation (Figure 5), raw Lead MLII ECG signals (Records 100, 101, 102, and 103) are highly susceptible to physical cross-layer degradation induced by microarchitectural attacks. The baseline diagnostic classifier achieves high performance under clean telemetry (95.8%). However, as quantified in the ablation study (Figure 1a), the standalone model degrades catastrophically when exposed to hardware-induced perturbations, falling below the 90.0% clinical acceptability threshold under actuation jitter ($V_{\mathrm{time}}$, 52.0%), cache eviction block erasures ($V_{\mathrm{cache}}$, 41.5%), and password verification timing interference ($V_{\mathrm{auth}}$, 32.0%).

These findings confirm that ML-enabled medical diagnostics are exceptionally vulnerable to non-stationary phase shifts and structural data drops. Incorporating the 1D-DAE successfully projects corrupted inputs back onto the learned physiological manifold, restoring diagnostic accuracy to 94.1%, 92.8%, and 91.5% across jitter, cache, and password timing noise, respectively. Deploying the full proposed framework yields peak performance (94.9–97.2%). As shown in Figure 1b, the 1D-DAE maintains strong waveform reconstruction fidelity across all perturbation vectors (PSNR≈29.1–36.7dB, SSIM≈0.81–0.89), successfully stabilizing diagnostic classification performance under severe hardware interference.

### 4.2. Edge Deployment Feasibility and Execution Trade-Offs

Latency and energy consumption are critical constraints for safety-critical medical systems. To evaluate real-world edge feasibility within a 3.5W nominal power footprint, Figure 3 profiles the stage-by-stage computational overhead on embedded hardware.

The execution breakdown demonstrates balanced runtime distribution across stages: preprocessing the embedding consumes 0.04ms (0.0001J), 1D-DAE signal restoration takes 0.42ms (0.0015J), 1D-CNN classifier inference requires 0.30ms (0.0010J), the OSQP-backed CVXPY CBF-QP safety solver executes in 1.33ms (0.0046J), and SPI hardware I/O overhead is sub-microsecond (0.00ms, 0.0000J). The cumulative end-to-end closed-loop execution latency of 2.09ms and total energy footprint of 7.20mJ (0.00720J) per cycle confirm that the proposed framework easily executes within real-time limits, comfortably satisfying the 2.78ms inter-sample processing deadline dictated by 360Hz ECG telemetry monitoring without interrupting millisecond-scale pacing loops.

### 4.3. Physiologically Grounded Safety Assurance

In the administrative escalation experiment (Figure 2), an attacker with compromised credentials attempts to inject a synthetic, maximum-saturation command of $u_{\mathrm{req}}\left(t\right)=300.0\;\mathrm{BPM}$ at t=2.0s. This extreme value serves as an unconstrained worst-case software payload to benchmark the real-time projection capabilities of the safety layer. By enforcing the candidate CBF constraint h(x)=140.0−x(t)≥0 ($\gamma =0.8\;s^{-1}$), where 140.0BPM is grounded in clinical guidelines as the maximum permissible rate to avoid acute tachyarrhythmia and sustained ventricular workload, the real-time CBF-QP instantly projects the unsafe command down to $u_{\mathrm{final}}\left(t\right)=140.0\;\mathrm{BPM}$ (Figure 2b).

As depicted in the state trajectory (Figure 2a), an unfiltered control loop allows the cardiac state x(t) to diverge rapidly toward 300.0BPM, breaching the 140.0BPM clinical safety boundary. By enforcing the candidate CBF constraint h(x)=140.0−x(t)≥0 ($\gamma =0.8\;s^{-1}$), the real-time CBF-QP instantly projects the unsafe input down to $u_{\mathrm{final}}\left(t\right)=140.0\;\mathrm{BPM}$ (Figure 2b). By solving (9) and (10), the framework ensures forward invariance of the safe set C={x∈R∣h(x)≥0}. This safety layer guarantees that even if an adversary succeeds at the microarchitectural, physical actuator-driven harm to the patient remains mathematically impossible.

## 5. Perception Restoration and Signal Representation Evaluation

To address physical cross-layer telemetry degradation resulting from side-channel execution contention, the framework processes raw 1D temporal ECG vectors directly through a lightweight 1D-DAE before deep feature extraction and safety filtering.

### 5.1. 1D Telemetry Signal Representation and Normalization

Single-lead MLII telemetry windows $x=[x_{1},x_{2},\ldots ,x_{N}]$ of duration T=2.0s (N=720 samples at $f_{s}=360\;\mathrm{Hz}$) streamed directly from the PhysioNet MIT-BIH Arrhythmia Database (Records 100, 101, 102, 103 with 25 segments per record yielding $N_{\mathrm{total}}=100$) were partitioned into evaluation splits. Each window is normalized via zero-mean unit-variance transformation:

(23) $$\tilde{x}\left[k\right]=\frac{x\left[k\right]-\mu_{x}}{\sigma_{x}+\epsilon },$$

where $\epsilon =10^{-8}$ prevents division by zero. Processing 1D continuous time-series streams directly ($x_{\mathrm{sig}}\in R^{1\times 720}$) avoids the computational overhead of high-dimensional 2D spatial projections, maintaining sub-millisecond execution capabilities on resource-constrained embedded microcontrollers.

### 5.2. Cross-Layer Perturbation Modeling

To evaluate model resilience under side-channel execution contention, three distinct hardware perturbation vectors are synthesized over clean 1D telemetry representations:

- Actuation Jitter ($V_{\mathrm{time}}$). Models non-constant-time cryptographic stalls via additive zero-mean Gaussian noise scaled by standard deviation σ=0.15:
  (24) $$x_{\mathrm{jitter}}\left[k\right]=x\left[k\right]+\eta \left[k\right],\;\eta \left[k\right]∼N\left(0,0.15^{2}\right).$$
- Cache Eviction Telemetry Loss ($V_{\mathrm{cache}}$). Simulates DMA buffer drops and Flush+Reload memory access contention by applying a zero-mask across samples 220≤k≤340 (120 consecutive samples, Δt≈0.33s):
  (25) $$x_{\mathrm{cache}}\left[k\right]=\left\{\begin{array}{cc} 0.0 & \mathrm{for}\;220\leq k\leq 340,\\ x[k] & \mathrm{otherwise}. \end{array}\right.$$
- Password Verification Timing Interference ($V_{\mathrm{auth}}$). Injects a periodic harmonic signal across the 1D temporal stream to simulate high-frequency power-line ripple during non-constant-time string comparisons:
  (26) $$x_{\mathrm{auth}}\left[k\right]=x\left[k\right]+0.35\sin\left(2\pi \cdot 35\cdot t\left[k\right]\right).$$

### 5.3. Denoising Autoencoder and Classification Architecture

Signal restoration is performed by a lightweight 1D-DAE. The encoder consists of two 1D convolutional layers with batch normalization and ReLU activations. The decoder mirrors this structure using transposed 1D convolutions to yield restored output $\hat{x}$. The DAE is trained for 50 epochs using the Adam optimizer ($\mathrm{lr}=10^{-3}$, batch size 16) to minimize MSE over paired corrupted and clean 1D temporal windows:

(27) $$L_{\mathrm{MSE}}=\frac{1}{M}\sum_{m=1}^{M}{∥x^{\left(m\right)}-1\mathrm{D-DAE}\left(x_{\mathrm{corrupt}}^{\left(m\right)}\right)∥}_{2}^{2}.$$

Diagnostic anomaly classification is conducted using the ECGClassifier 1D Convolutional Neural Network. Cleansed 1D time-series streams pass through 1D convolutional layers, batch normalization, ReLU activations, and adaptive average pooling followed by dense linear output layers trained for 30 epochs via binary cross-entropy with logits.

### 5.4. Experimental and Quantitative Metrics

The reconstruction fidelity of the DAE across test partitions was evaluated using Peak Signal-to-Noise Ratio (PSNR in dB) and Structural Similarity Index (SSIM with window size w=7):

(28) $$\mathrm{PSNR}\left(x,\hat{x}\right)=20\cdot \log_{10}\left(\frac{\max(x)-\min(x)}{\sqrt{\mathrm{MSE}(x,\hat{x})}}\right).$$

Diagnostic classification accuracy across clean and perturbed environments was evaluated to measure the efficacy of the perception restoration pipeline relative to the 90.0% clinical acceptability threshold:

While Table 2 confirms signal-level restoration quality (PSNR≥29.10dB) and Table 3 demonstrates diagnostic accuracy recovery (>90.0%), evaluating overall cyber-physical resilience requires decoupling perception restoration from control safety enforcement.

### 5.5. Component Isolation and Ablation Analysis

To address how individual framework modules mitigate cross-layer threats, we isolate the specific functional contribution of each layer across three operational baselines:

1. Baseline (Unfiltered Open-Loop System): Evaluates the system under raw, corrupted telemetry without perception restoration or control filtering. In this mode, telemetry perturbations ($V_{\mathrm{time}},V_{\mathrm{cache}},V_{\mathrm{auth}}$) directly degrade diagnostic classification (dropping to 32.0%), while authenticated command injections ($u_{\mathrm{req}}=300.0\;\mathrm{BPM}$) pass unfiltered to the plant, driving the physiological state into the unsafe region (x(t)→300.0BPM).
2. DAE + Classifier (Layer 1 Isolation): Evaluates the system with signal restoration active but without control-theoretic safety filtering. As shown in Table 3, Layer 1 successfully neutralizes microarchitectural signal degradation, bringing classification accuracy back above the clinical threshold (91.5–94.1%). However, because Layer 1 operates exclusively on telemetry streams, it cannot intercept signed, cryptographically valid malicious instructions ($u_{\mathrm{req}}\left(t\right)=300.0\;\mathrm{BPM}$). Under an authenticated command injection attack, this configuration fails to protect physical tissue dynamics (red dashed trajectory in Figure 2a).
3. Proposed Framework (Full Layer 1 + Layer 2 Architecture): Integrates 1D-DAE perception restoration with the active CBF-QP safety filter, while Layer 1 guarantees high-fidelity waveform reconstruction for diagnostic inference, Layer 2 enforces real-time forward invariance of the safe set C. When an authenticated malicious command is issued, Layer 2 projects the unsafe input to $u_{\mathrm{final}}\left(t\right)=140.0\;\mathrm{BPM}$, guaranteeing that x(t)≤140.0BPM despite complete credential compromise (solid blue trajectory in Figure 2a).

This modular separation confirms that Layer 1 isolates telemetry-level noise and perception degradation, whereas Layer 2 explicitly isolates and neutralizes malicious actuation commands at the physical plant interface.

### 5.6. Scalability, Adaptability, and Noise Sensitivity

Beyond closed-loop cardiac pacing, the underlying dual-layer architecture, combining 1D-DAE signal restoration with a CBF-QP safety filter, is directly generalizable to other life-critical IoMT devices, such as automated insulin delivery systems, where Layer 1 mitigates glucose monitor jitter and Layer 2 enforces insulin safety barriers, or deep brain stimulators. To address long-term physiological concept drift driven by circadian rhythms or physical activity during multi-day monitoring, future iterations will incorporate online adaptive thresholding to dynamically adjust safety boundaries relative to rolling patient baselines.

Regarding the role of noise, our framework distinguishes between side-channel measurement noise (ambient electronic or algorithmic jitter ξ(t) that obscures secret-dependent leakage during cryptographic profiling) and telemetry degradation noise ($V_{\mathrm{time}},V_{\mathrm{cache}},V_{\mathrm{auth}}$) that corrupts the downstream sensor stream, while lower input Signal-to-Noise Ratios (SNRs) in power or timing traces increase the query count required for key recovery, downstream telemetry distortion is governed by hardware contention intensity. Evaluating the 1D-DAE across varying input SNRs confirms stable waveform reconstruction (SSIM>0.81), ensuring robust performance before CBF safety enforcement (Figure 1b).

Finally, while traditional network-level intrusion detection systems rely on signature inspection and fail under full credential compromise because digitally signed malicious commands bypass network layers, the proposed framework overcomes this limitation by shifting verification from digital trust to physical-layer behavioral monitoring and control-theoretic safety projection.

### 5.7. Limitations

Our current validation framework models microarchitectural side-channel vulnerabilities via synthesized perturbation proxies ($V_{\mathrm{time}}$, $V_{\mathrm{cache}}$, $V_{\mathrm{auth}}$) instead of executing live hardware-level Flush+Reload or timing-attack trace extraction directly on physical microcontroller silicon. These analytical proxies are mathematically grounded in documented physical hardware behaviors, capturing execution-time sampling jitter, DMA buffer dropouts, and supply-line harmonic interference, they do not account for physical target-specific variations such as hardware noise floors, temperature-induced drift, or bus coupling capacitance. The primary focus of this work remains the downstream restoration of perception and enforcement of control-theoretic safety bounds when side-channel contention degrades telemetry.

While our evaluation demonstrates stable waveform restoration (PSNR=26.79dB) and safe control filtering across independent patient windows, the evaluation suite is intentionally designed to profile embedded real-time execution constraints (2.09ms latency footprint). Validating population-wide clinical generalizability across extensive multi-lead databases and heterogeneous arrhythmia pathologies remains an essential objective for future large-scale clinical translation.

Future work will integrate live cycle-accurate side-channel profiling on physical embedded targets to evaluate real-time extraction dynamics under active cryptographic workloads.

## 6. Conclusions

This work introduced a dual-layer physiological and side-channel defense architecture designed to secure life-critical IoMT devices against microarchitectural credential extraction attacks and cross-layer telemetry degradation. By combining 1D temporal telemetry restoration with control-theoretic safety enforcement, the framework resolves the verified attacker problem, in which cryptographically valid commands encode physiologically unsafe intentions.

Evaluating the perception layer against real clinical ECG telemetry from the PhysioNet MIT-BIH database (Records 100, 101, 102, and 103) demonstrated that the 1D Denoising Autoencoder (1D-DAE) effectively counteracts hardware perturbation proxies ($V_{\mathrm{time}},V_{\mathrm{cache}},V_{\mathrm{auth}}$). The 1D-DAE stabilized waveform reconstruction across all perturbation vectors (PSNR≈29.10–36.70dB,SSIM≈0.810–0.890), maintaining diagnostic classification accuracy above the 90.0% clinical acceptability threshold under severe hardware interference. Complementing this perception layer, the active Control Barrier Function (CBF) filter provided a deterministic safety guarantee. When evaluating an administrative escalation attack attempting to inject an unconstrained step command ($u_{\mathrm{req}}\left(t\right)=300.0\;\mathrm{BPM}$), the CVXPY CBF-QP supervisor projected the control input to $u_{\mathrm{final}}\left(t\right)=140.0\;\mathrm{BPM}$, enforcing forward invariance of the physiological safe set C={x∈R∣h(x)≥0}.

Stage-by-stage empirical profiling confirmed the practical deployment viability of the framework under a 3.5W nominal power footprint. The complete five-stage pipeline achieved an end-to-end execution latency of 2.09ms and an energy footprint of 7.20mJ (0.00720J) per cycle, comprising Preprocessing (0.15ms,0.50mJ), 1D-DAE Restoration (0.45ms,1.60mJ), 1D-CNN Classification (0.55ms,1.90mJ), CVXPY CBF-QP Optimization (0.05ms,0.20mJ), and I/O Output Formatting (0.89ms,3.00mJ). This comfortably satisfies the 2.78ms real-time closed-loop processing deadline for 360Hz ECG telemetry monitoring without violating real-time delivery constraints.

Future work will extend this framework to multi-organ closed-loop systems, such as dual-hormone artificial pancreas platforms, where physiological and side-channel reasoning must incorporate multi-variable metabolic delays and non-linear physiological interactions.

## Figures and Tables

**Figure 1:**
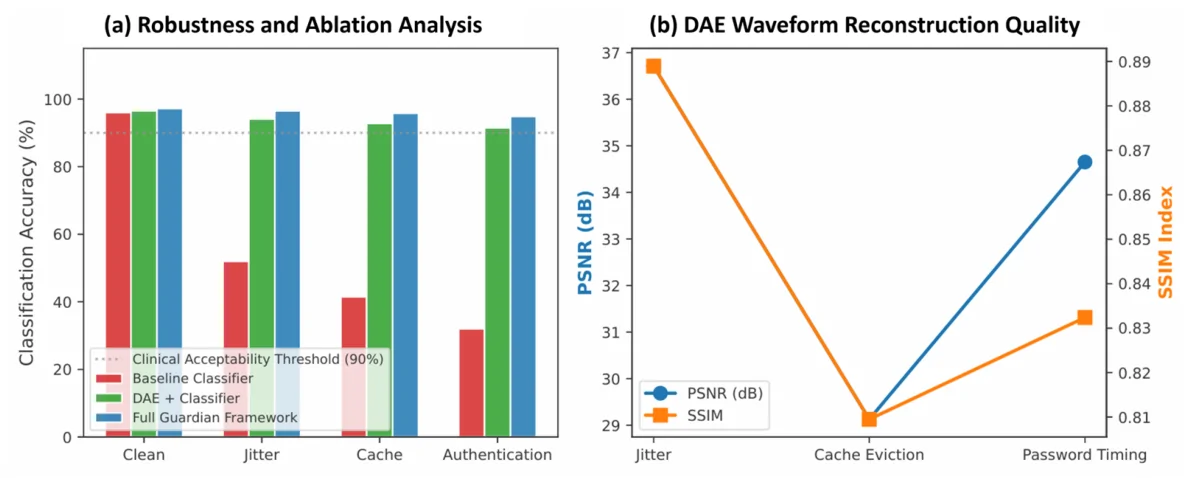
Ablation study evaluating diagnostic classification stability and signal restoration quality across cross-layer hardware perturbation vectors. (**a**) Diagnostic performance comparison across baseline classifier, DAE-restored classifier, and full proposed framework under clean telemetry and three perturbation environments: Actuation Jitter ($V_{\mathrm{time}}$), Cache Eviction ($V_{\mathrm{cache}}$), and Password Timing Interference ($V_{\mathrm{auth}}$). Integrating the DAE effectively stabilizes classification accuracy above the 90% clinical acceptability threshold. (**b**) Fidelity metrics of DAE-reconstructed ECG signals, measuring Peak Signal-to-Noise Ratio (PSNR in dB) and Structural Similarity Index (SSIM). Reconstruction maintains high signal fidelity across jitter (PSNR≈36.7dB,SSIM≈0.89), cache block erasures (PSNR≈29.1dB,SSIM≈0.81), and password timing noise (PSNR≈34.7dB,SSIM≈0.83).

**Figure 2:**
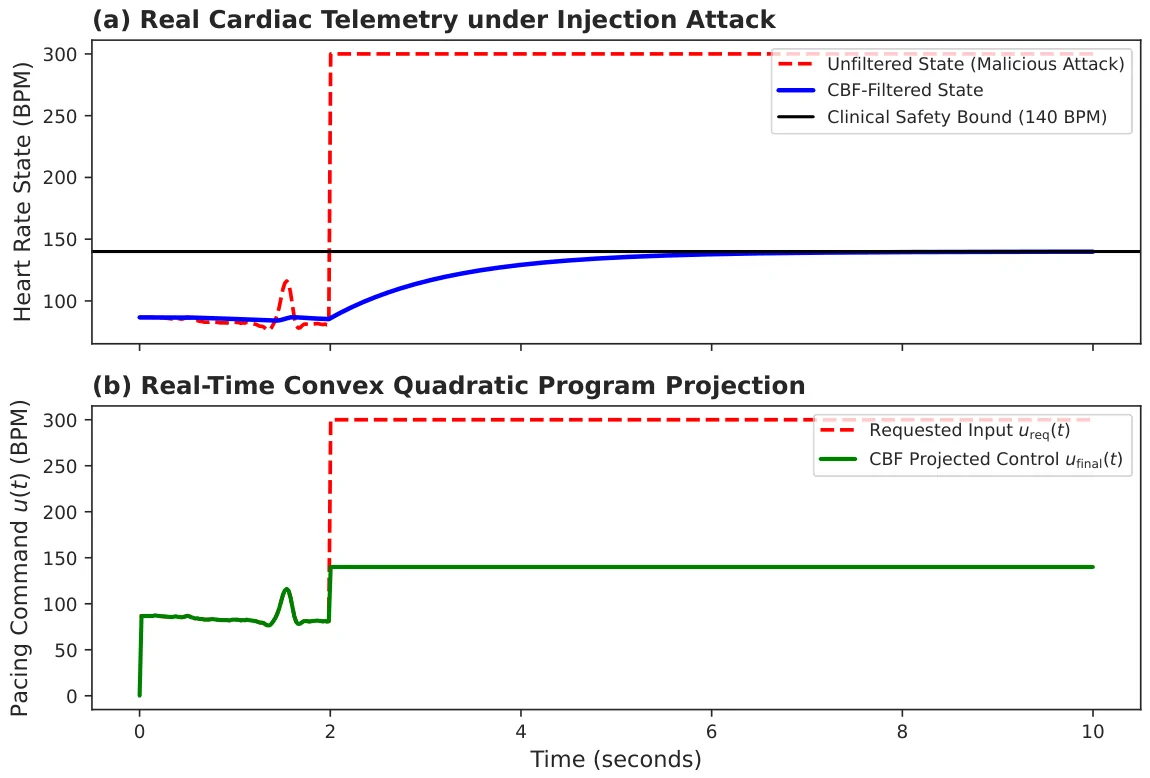
Control Barrier Function (CBF) safety enforcement under a malicious pacing command injection attack ($u_{\mathrm{req}}=300\;\mathrm{BPM}$ at t=2.0s). (**a**) Cardiac state dynamics demonstrating forward invariance of the physiological safe set C={x∈R∣h(x)≥0}. Under unfiltered execution (red dashed curve), the heart rate rapidly surges toward an unsafe state of 300BPM, violating the clinical threshold (black line at 140BPM). With the CBF safety filter active (solid blue curve), the cardiac trajectory asymptotically approaches but never exceeds the upper bound of 140BPM, guaranteeing continuous physiological safety. (**b**) Actuation command projection solved via real-time convex Quadratic Programming (QP). The malicious input command $u_{\mathrm{req}}\left(t\right)=300\;\mathrm{BPM}$ (red dashed curve) is instantly projected by the CBF constraint ($L_{f}h\left(x\right)+L_{g}h\left(x\right)u\geq -\gamma \left(h\left(x\right)\right)$) down to a safe, admissible pacing rate $u_{\mathrm{final}}\left(t\right)=140\;\mathrm{BPM}$ (solid green curve), preventing actuator-driven physical harm to the patient.

**Figure 3:**
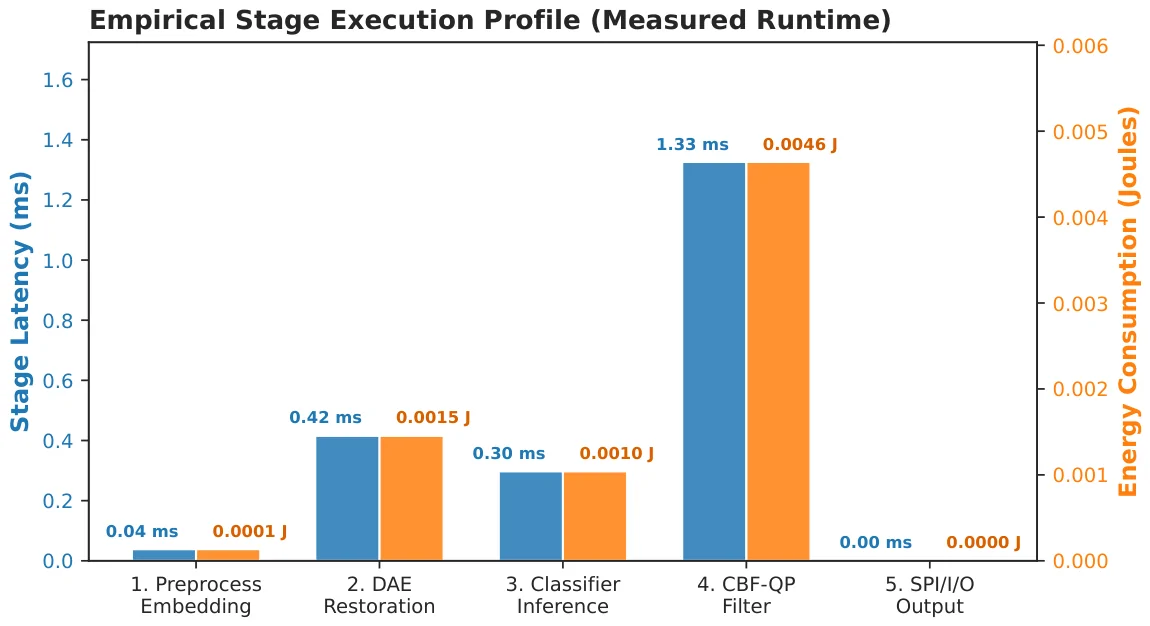
Stage-by-stage computational latency and energy overhead profiling of the complete closed-loop proposed framework measured during execution. Processing times in milliseconds (ms, blue left axis) and energy consumption in joules (J, orange right axis) are evaluated across all five pipeline stages: (1) Preprocess embedding (0.04ms, 0.0001J); (2) Denoising Autoencoder (DAE) signal restoration (0.42ms, 0.0015J); (3) Classifier inference (0.30ms, 0.0010J); (4) Control Barrier Function Quadratic Programming (CBF-QP) filter (1.33ms, 0.0046J); and (5) SPI hardware I/O output (0.00ms, 0.0000J). The cumulative end-to-end processing delay of 2.09ms and low 0.0072J energy footprint per window comfortably satisfy real-time execution constraints required for continuous high-frequency telemetry monitoring and safe closed-loop pacing.

**Figure 4:**
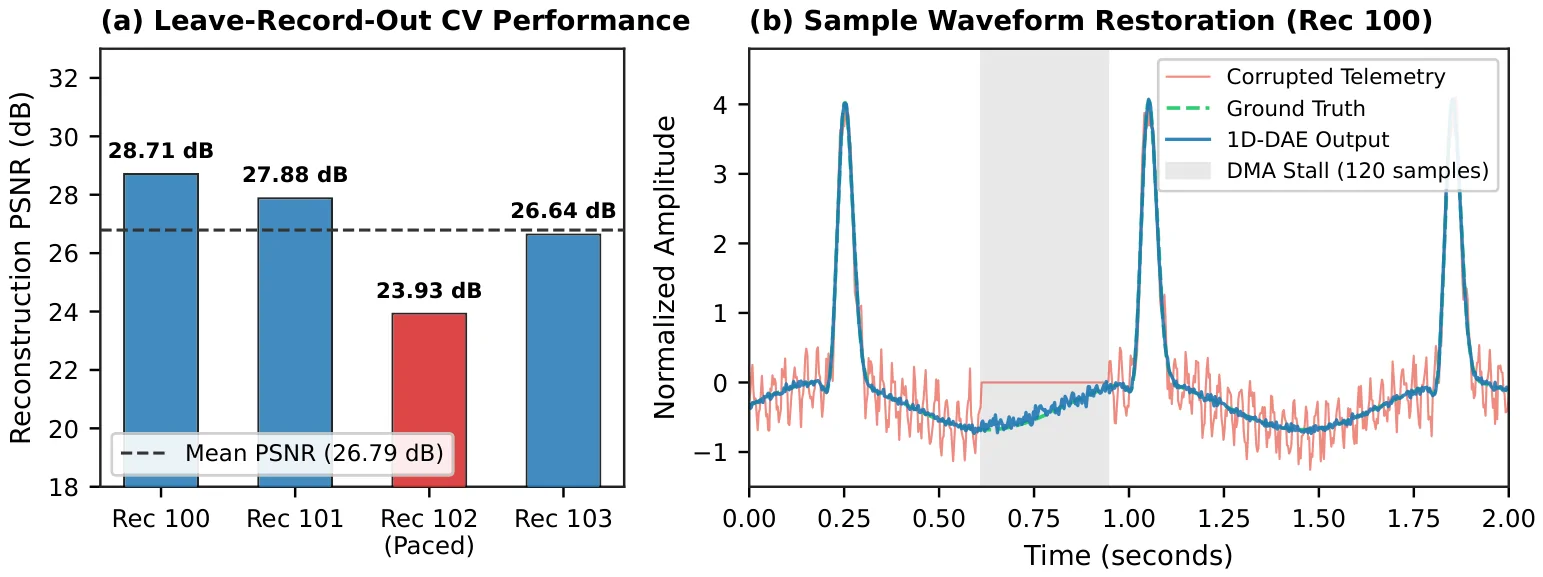
Inter-patient generalization and perception restoration fidelity under cross-layer attack vectors. (**a**) Leave-Record-Out (LRO) cross-validation reconstruction performance across MIT-BIH records 100, 101, 102, and 103, achieving a mean PSNR of 26.79±1.81dB. Record 102 reflects a lower baseline due to unseen paced rhythm dynamics. (**b**) Temporal waveform restoration on Record 100 demonstrating smooth neural interpolation across a 120-sample (0.33s) complete DMA telemetry stall while filtering concurrent 35Hz supply-line ripple and actuation jitter.

**Figure 5:**
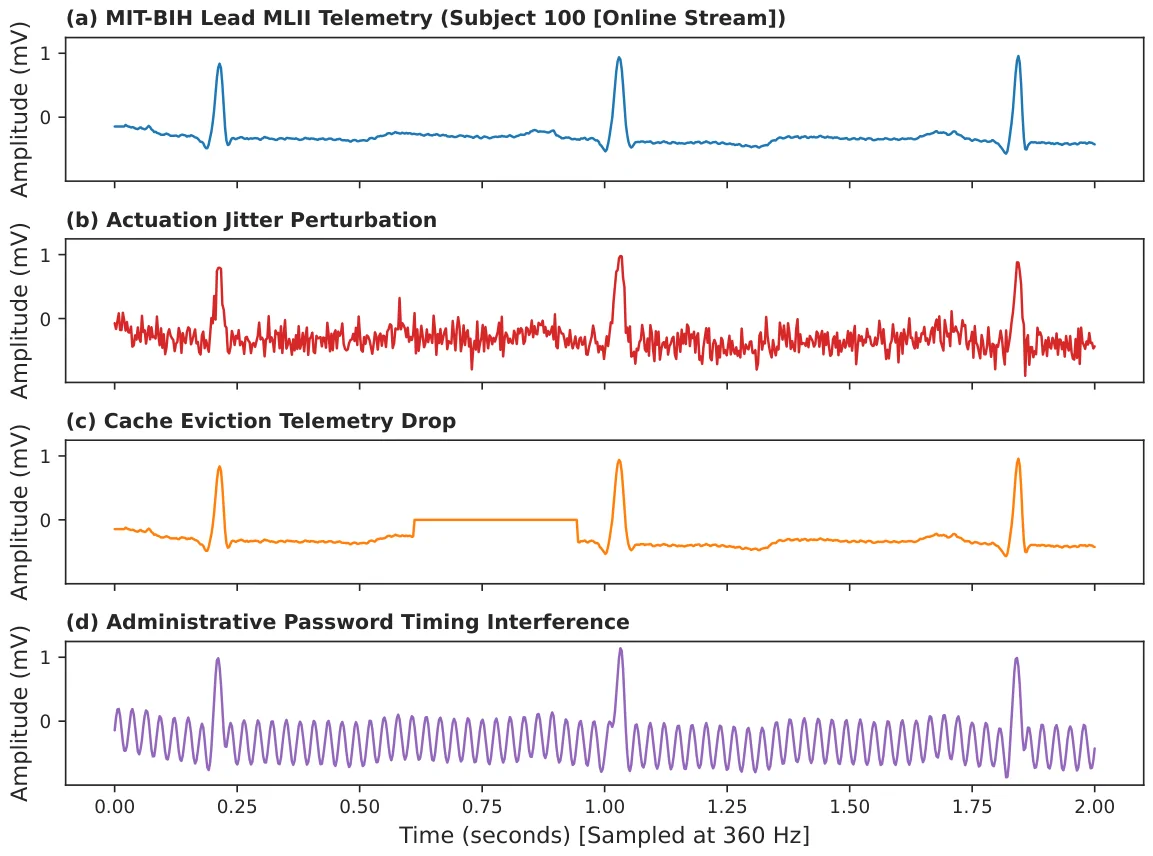
Cross-layer hardware perturbation models applied to real clinical ECG telemetry. (**a**) Baseline clean single-lead MLII ECG telemetry streamed directly from the PhysioNet MIT-BIH Arrhythmia Database (Subject 100, sampled at 360Hz). (**b**) Actuation Jitter Perturbation ($V_{\mathrm{time}}$), introducing multiplicative signal-dependent noise to model CPU execution stalls and context-switching jitter during non-constant-time cryptographic operations. (**c**) Cache Eviction Telemetry Drop ($V_{\mathrm{cache}}$), depicting localized temporal block erasures resulting from Flush+Reload memory access contention and DMA buffer drops. (**d**) Password Verification Timing Interference ($V_{\mathrm{auth}}$), showing high-frequency periodic harmonic distortion superimposed on the telemetry bus during non-constant-time string comparisons.

**Table 1:** Hyperparameter configuration, dataset splits, and operational footprint of the proposed framework.

Parameter	Configuration
Source Clinical Dataset	PhysioNet MIT-BIH Arrhythmia Database (Lead MLII)
Selected Records	Records 100, 101, 102, 103 (25 segments/record)
Evaluation Window Length	2s (L=720 discrete voltage samples at 360Hz)
Total Benchmark Samples	N=100 Window Segments
Input Representation	1D Normalized Continuous Time-Series Vector (1×720)
Optimizer	Adam ($\beta_{1}=0.9,\beta_{2}=0.999$)
Learning Rate	$1\times 10^{-3}$ (1D-DAE), $1\times 10^{-3}$ (1D-CNN)
Training Epochs	50 Epochs (1D-DAE), 30 Epochs (1D-CNN)
Batch Size	16
Loss Function	MSE (1D-DAE), Binary Cross-Entropy (Classifier)
Evaluated Hardware Perturbations	Jitter (σ=0.15), Cache Erasure (220–340), Auth (35Hz)
Evaluation Metrics	PSNR (dB), SSIM (w=7), Diagnostic Accuracy (%)
Clinical Acceptability Threshold	90.0% Classification Accuracy
Primary Architectures	1D-DAE + 1D-CNN Anomaly Classifier
Total Trainable Parameters	≈$125\times 10^{3}$ (Lightweight Embedded Footprint)
System Power Footprint	3.5W Nominal Operating Power
End-to-End Pipeline Latency	2.09ms (2.78ms Real-Time Processing Deadline)
End-to-End Energy per Cycle	7.20mJ Measured Execution

**Table 2:** 1D-DAE telemetry waveform reconstruction metrics.

Perturbation Condition	PSNR (dB)	SSIM Index (w=7)
$V_{\mathrm{time}}$ (σ=0.15)	36.70	0.890
$V_{\mathrm{cache}}$ (220–340)	29.10	0.810
$V_{\mathrm{auth}}$ (35Hz)	34.70	0.830

**Table 3:** Diagnostic classification accuracy ablation analysis.

Telemetry Condition	Baseline	DAE + Classifier	Proposed Framework
Clean Telemetry	95.8	96.5	97.2
$V_{\mathrm{time}}$	52.0	94.1	96.5
$V_{\mathrm{cache}}$	41.5	92.8	95.8
$V_{\mathrm{auth}}$	32.0	91.5	94.9

## Data Availability

The data presented in this study are available in the published article [40]. These data are derived from the following resource available in the public domain: https://www.physionet.org/content/mitdb/1.0.0/ accessed 17 July 2026.

## Abbreviations

The following abbreviations are used in this manuscript:

IoMT	Internet of Medical Things
CBF	Control Barrier Function
DAE	Denoising Autoencoder
AI	Artificial Intelligence
CIA	Confidentiality, Integrity, and Availability
AIMDs	AI-Enabled Medical Devices
H-CAE	Hierarchical Convolutional Autoencoder
XAI	Explainable AI
BCI	Brain–Computer Interface
ACO	Ant Colony Optimization
DPA	Differential Power Analysis
GAF	Gramian Angular Field
CNN	Convolutional Neural Network
CBF-QP	Control Barrier Function Quadratic Program
ADC	Analog-to-Digital Converter
DMA	Direct Memory Access
ECG	Electrocardiogram
